# (1*S*,3*R*,8*R*)-2,2-Di­chloro-3,7,7,10-tetra­methyl­tri­cyclo­[6.4.0.0^1,3^]dodec-9-en-11-one

**DOI:** 10.1107/S1600536813011781

**Published:** 2013-05-04

**Authors:** Naja Ourhriss, Ahmed Benharref, Abdelouahd Oukhrib, Jean-Claude Daran, Moha Berraho

**Affiliations:** a Laboratoire de Chimie des Substances Naturelles, "Unité Associé au CNRST (URAC16)", Faculté des Sciences Semlalia, BP 2390 Bd My Abdellah, 40000 Marrakech, Morocco; b Laboratoire de Chimie de Coordination, 205 route de Narbonne, 31077 Toulouse Cedex 04, France

## Abstract

The title compound, C_16_H_22_Cl_2_O, was synthesized from β-himachalene (3,5,5,9-tetra­methyl-2,4a,5,6,7,8-hexa­hydro-1*H*-benzo­cyclo­heptene), which was isolated from the essential oil of the Atlas cedar (*Cedrus Atlantica*). The mol­ecule is built up from fused six- and seven-membered rings and an additional three-membered ring arising from the reaction of himachalene with di­chloro­carbene. The six-membered ring has an envelope conformation, with the C atom belonging to the three-membered ring forming the flap, whereas the seven-membered ring displays a screw-boat conformation; the dihedral angle between the rings (all atoms) is 59.65 (14)°.

## Related literature

For background to the essential oil of the Alas cedar (*Cedrus atlantica*), see: Joseph & Dev (1968 ▶); Plattier & Teiseire (1974 ▶). For the reactivity and biological properties of β-himachalene, see: Benharref *et al.* (2012 ▶); Chekroun *et al.* (2000 ▶); El Jamili *et al.* (2002 ▶); Lassaba *et al.* (1998 ▶); Dakir *et al.* (2004 ▶); Daoubi *et al.* (2004 ▶). For conformational analysis, see: Cremer & Pople (1975 ▶).
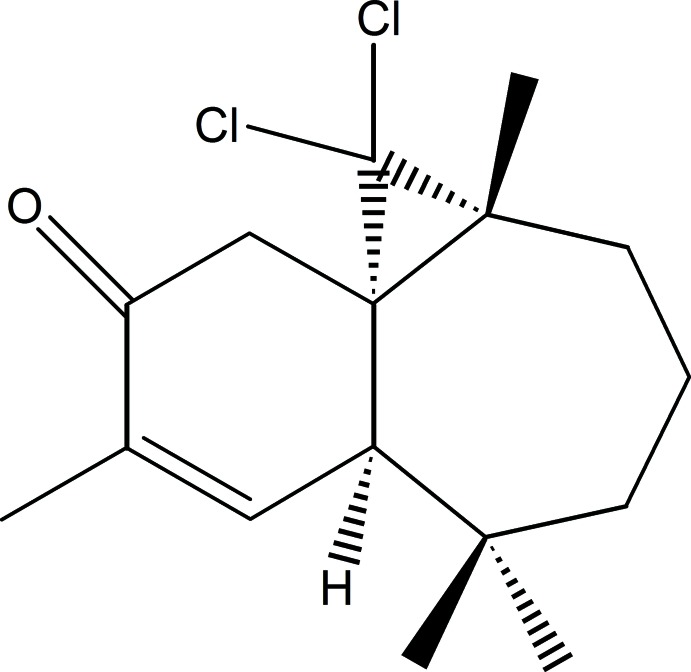


## Experimental

### 

#### Crystal data

C_16_H_22_Cl_2_O
*M* *_r_* = 301.24
Monoclinic, 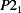

*a* = 8.8780 (3) Å
*b* = 10.3340 (3) Å
*c* = 8.9230 (3) Å
β = 108.805 (4)°
*V* = 774.94 (4) Å^3^
*Z* = 2
Cu *K*α radiation
μ = 3.67 mm^−1^
*T* = 180 K
0.30 × 0.25 × 0.21 mm

#### Data collection

Agilent Xcalibur (Eos, Gemini ultra) diffractometer
Absorption correction: multi-scan (*CrysAlis PRO*; Agilent, 2010 ▶) *T*_min_ = 0.761, *T*_max_ = 1.000
2787 measured reflections
1835 independent reflections
1779 reflections with *I* > 2σ(*I*)
*R*_int_ = 0.027
θ_max_ = 60.6°

#### Refinement

*R*[*F* ^2^ > 2σ(*F* ^2^)] = 0.028
*wR*(*F* ^2^) = 0.065
*S* = 1.03
1835 reflections
176 parameters
1 restraint
H-atom parameters constrained
Δρ_max_ = 0.16 e Å^−3^
Δρ_min_ = −0.17 e Å^−3^
Absolute structure: Flack & Bernardinelli (2000 ▶), 614 Friedel pairs
Flack parameter: 0.014 (15)

### 

Data collection: *CrysAlis PRO* (Agilent, 2010 ▶); cell refinement: *CrysAlis PRO*; data reduction: *CrysAlis PRO*; program(s) used to solve structure: *SHELXS97* (Sheldrick, 2008 ▶); program(s) used to refine structure: *SHELXL97* (Sheldrick, 2008 ▶); molecular graphics: *ORTEP-3 for Windows* (Farrugia, 2012 ▶) and *PLATON* (Spek, 2009 ▶); software used to prepare material for publication: *WinGX* (Farrugia, 2012 ▶).

## Supplementary Material

Crystal structure: contains datablock(s) I, global. DOI: 10.1107/S1600536813011781/tk5222sup1.cif

Structure factors: contains datablock(s) I. DOI: 10.1107/S1600536813011781/tk5222Isup2.hkl

Supplementary material file. DOI: 10.1107/S1600536813011781/tk5222Isup3.cml

Additional supplementary materials:  crystallographic information; 3D view; checkCIF report

## Figures and Tables

**Fig. 1.**
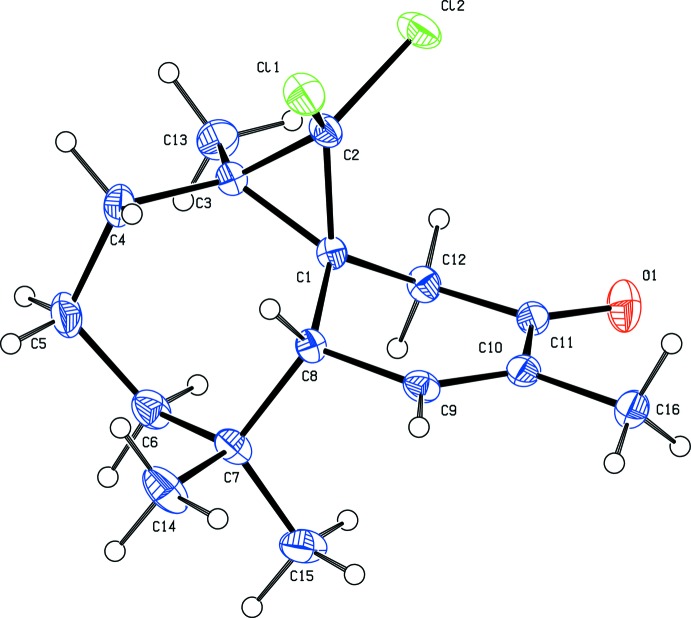
Molecular structure of the title compound with the atom-labelling scheme. Displacement ellipsoids are drawn at the 30% probability. level. H atoms are represented as small spheres of arbitrary radii.

## supplementary crystallographic information

### Comment

The essential oil of the Alas cedar (*Cedrus atlantica*) consist mainly (50%) of a bicyclic hydrocarbon called essential oil of the Alas cedar (*Cedrus atlantica*) consist mainly (50%) of a bicyclic hydrocarbon called (Joseph & Dev, 1968; Plattier & Teiseire, 1974). The reactivity of this sesquiterpene and its derivatives has been studied extensively by our team in order to prepare new products having biological proprieties (Lassaba *et al.*, 1998; Chekroun *et al.*, 2000; El Jamili *et al.*, 2002; Dakir *et al.*, 2004; Benharref *et al.* 2012). Indeed, these compounds were tested, using the food poisoning technique, for their potential antifungal activity against phytopathogen *Botrytis cinerea* (Daoubi *et al.*, 2004). We present here the crystal structure of the title compound, (1*S*,3*R*,8*R*)-2,2-dichloro-3,7,7,10-tetramethyltricyclo [6.4.0.0^1^,^3^]dodec-9-en-10-one. The molecule is built up from two fused six-and seven- membered rings and an additional three-membered ring from the reaction with the carbene (Fig. 1). The six-membered ring has an envelope conformation, as indicated by the total puckering amplitude QT = 0.453 (3) Å and spherical polar angle θ = 123.4 (4)° with φ = 170.0 (4)°, whereas the seven-membered ring display a boat conformation with QT = 1.1545 (3) Å, θ = 87.74 (2)°, φ2 = -48.13 (14)° and φ3 = -134.45 (4)° (Cremer & Pople, 1975). Owing to the presence of Cl atoms, the absolute configuration could be fully confirmed, by refining the Flack parameter (Flack & Bernardinelli, 2000) as C1(*S*), C3(*R*) and C8(*R*).

### Experimental

In a reactor containing a solution of (1*S*, 3*R*, 8*R*)-2,2- dichloro-3,7,7,10 tetramethyltricyclo [6.4.0.0^1^,^3^] dodec-9-ene (1 g, 3.48 mmol) (El Jamili *et al.*, 2002) in 50 ml tetrahydrofuran and water (THF/H_2_O) (4:1) cooled to 273 K and kept in the dark, was added in small portions 1.23 g (6.96 mmol) of *N*-bromosccinimide (NBS). The reaction mixture was left stirring for 1 h, after which 20 ml of a saturated solution of NaHCO_3_ was added. Subsequently, the extraction was performed three times with diethyl ether (3x 20 ml). The organic extracts were dried over Na_2_SO_4_, filtered, concentrated, and chromatographed. The title compound was obtained with a yield of 80% and was recrystallized from its hexane solution.

### Refinement

All H atoms were fixed geometrically and treated as riding with C—H = 0.96 Å (methyl),0.97 Å (methylene), 0.98 Å (methine) with *U*_iso_(H) = 1.2*U*_eq_(methylene, methine) or *U*_iso_(H) = 1.5*U*_eq_(methyl). Owing to the tiny size of the crystal and to define the correct absolute structure determination, the data were collected using Cu radiation.

### Crystal data

**Table:**

C_16_H_22_Cl_2_O	*F*(000) = 320
*M**_r_* = 301.24	*D*_x_ = 1.291 Mg m^−^^3^
Monoclinic, *P*2_1_	Cu *K*α radiation, λ = 1.5418 Å
Hall symbol: P 2yb	Cell parameters from 1955 reflections
*a* = 8.8780 (3) Å	θ = 4.3–60.5°
*b* = 10.3340 (3) Å	µ = 3.67 mm^−^^1^
*c* = 8.9230 (3) Å	*T* = 180 K
β = 108.805 (4)°	Block, colourless
*V* = 774.94 (4) Å^3^	0.30 × 0.25 × 0.21 mm
*Z* = 2	

### Data collection

**Table:**

Agilent Xcalibur (Eos, Gemini ultra) diffractometer	1835 independent reflections
Radiation source: Enhance Ultra (Cu) X-ray Source	1779 reflections with *I* > 2σ(*I*)
Mirror monochromator	*R*_int_ = 0.027
Detector resolution: 16.1978 pixels mm^-1^	θ_max_ = 60.6°, θ_min_ = 5.2°
ω scans	*h* = −9→8
Absorption correction: multi-scan (*CrysAlis PRO*; Agilent, 2010)	*k* = −11→11
*T*_min_ = 0.761, *T*_max_ = 1.000	*l* = −8→10
2787 measured reflections	

### Refinement

**Table:**

Refinement on *F*^2^	Secondary atom site location: difference Fourier map
Least-squares matrix: full	Hydrogen site location: inferred from neighbouring sites
*R*[*F*^2^ > 2σ(*F*^2^)] = 0.028	H-atom parameters constrained
*wR*(*F*^2^) = 0.065	*w* = 1/[σ^2^(*F*_o_^2^) + (0.0268*P*)^2^] where *P* = (*F*_o_^2^ + 2*F*_c_^2^)/3
*S* = 1.03	(Δ/σ)_max_ < 0.001
1835 reflections	Δρ_max_ = 0.16 e Å^−^^3^
176 parameters	Δρ_min_ = −0.17 e Å^−^^3^
1 restraint	Absolute structure: Flack & Bernardinelli (2000), 614 Friedel pairs
Primary atom site location: structure-invariant direct methods	Flack parameter: 0.014 (15)

### Special details

**Table:**

Geometry. All s.u.'s (except the s.u. in the dihedral angle between two l.s. planes) are estimated using the full covariance matrix. The cell s.u.'s are taken into account individually in the estimation of s.u.'s in distances, angles and torsion angles; correlations between s.u.'s in cell parameters are only used when they are defined by crystal symmetry. An approximate (isotropic) treatment of cell s.u.'s is used for estimating s.u.'s involving l.s. planes.
Refinement. Refinement of *F*^2^ against ALL reflections. The weighted *R*-factor *wR* and goodness of fit *S* are based on *F*^2^, conventional *R*-factors *R* are based on *F*, with *F* set to zero for negative *F*^2^. The threshold expression of *F*^2^ > 2σ(*F*^2^) is used only for calculating *R*-factors(gt) *etc*. and is not relevant to the choice of reflections for refinement. *R*-factors based on *F*^2^ are statistically about twice as large as those based on *F*, and *R*- factors based on ALL data will be even larger.

### Fractional atomic coordinates and isotropic or equivalent isotropic displacement parameters (Å^2^)

**Table:**

	*x*	*y*	*z*	*U*_iso_*/*U*_eq_	
Cl1	0.37702 (8)	0.87301 (6)	0.63481 (9)	0.04024 (19)	
Cl2	0.53172 (8)	0.67535 (7)	0.51438 (9)	0.0422 (2)	
O1	0.7317 (2)	0.4592 (2)	0.9131 (3)	0.0472 (6)	
C1	0.3527 (3)	0.6081 (2)	0.7144 (3)	0.0230 (6)	
C2	0.3817 (3)	0.7052 (2)	0.6004 (3)	0.0278 (6)	
C3	0.2321 (3)	0.6249 (2)	0.5493 (3)	0.0280 (6)	
C4	0.0763 (3)	0.6914 (3)	0.5429 (3)	0.0367 (7)	
H4A	0.0196	0.7190	0.4329	0.044*	
H4B	0.1000	0.7696	0.6105	0.044*	
C5	−0.0311 (3)	0.6006 (3)	0.5995 (4)	0.0417 (8)	
H5A	−0.1098	0.6536	0.6294	0.050*	
H5B	−0.0906	0.5446	0.5100	0.050*	
C6	0.0567 (3)	0.5144 (3)	0.7398 (3)	0.0366 (7)	
H6A	0.1202	0.4510	0.7025	0.044*	
H6B	−0.0241	0.4650	0.7708	0.044*	
C7	0.1685 (3)	0.5800 (3)	0.8897 (3)	0.0310 (6)	
C8	0.3075 (3)	0.6569 (3)	0.8553 (3)	0.0249 (6)	
H8	0.2677	0.7473	0.8285	0.030*	
C9	0.4529 (3)	0.6672 (3)	0.9982 (3)	0.0276 (6)	
H9	0.4453	0.7195	1.0830	0.033*	
C10	0.5923 (3)	0.6092 (3)	1.0171 (3)	0.0289 (7)	
C11	0.6079 (3)	0.5170 (3)	0.8973 (3)	0.0289 (6)	
C12	0.4618 (3)	0.4911 (2)	0.7555 (3)	0.0276 (6)	
H12A	0.4028	0.4166	0.7788	0.033*	
H12B	0.4953	0.4681	0.6633	0.033*	
C13	0.2113 (4)	0.5220 (3)	0.4243 (3)	0.0410 (7)	
H13A	0.1623	0.5602	0.3193	0.061*	
H13B	0.1426	0.4530	0.4411	0.061*	
H13C	0.3154	0.4858	0.4313	0.061*	
C14	0.0742 (4)	0.6748 (4)	0.9559 (4)	0.0483 (8)	
H14A	0.0328	0.7447	0.8794	0.072*	
H14B	0.1439	0.7114	1.0555	0.072*	
H14C	−0.0145	0.6294	0.9753	0.072*	
C15	0.2324 (4)	0.4722 (3)	1.0132 (3)	0.0422 (8)	
H15A	0.3011	0.5103	1.1123	0.063*	
H15B	0.2937	0.4101	0.9733	0.063*	
H15C	0.1429	0.4277	1.0326	0.063*	
C16	0.7361 (3)	0.6288 (3)	1.1612 (4)	0.0395 (8)	
H16A	0.8121	0.6856	1.1345	0.059*	
H16B	0.7863	0.5450	1.1974	0.059*	
H16C	0.7034	0.6686	1.2456	0.059*	

### Atomic displacement parameters (Å^2^)

**Table:**

	*U*^11^	*U*^22^	*U*^33^	*U*^12^	*U*^13^	*U*^23^
Cl1	0.0401 (4)	0.0234 (3)	0.0606 (5)	0.0026 (3)	0.0209 (3)	0.0072 (3)
Cl2	0.0418 (4)	0.0447 (4)	0.0518 (4)	0.0023 (3)	0.0313 (3)	0.0049 (4)
O1	0.0303 (11)	0.0442 (12)	0.0622 (14)	0.0111 (10)	0.0081 (9)	0.0002 (11)
C1	0.0221 (13)	0.0210 (12)	0.0259 (15)	0.0004 (10)	0.0077 (10)	−0.0016 (11)
C2	0.0249 (14)	0.0268 (14)	0.0358 (16)	0.0033 (10)	0.0153 (11)	0.0027 (12)
C3	0.0240 (14)	0.0314 (15)	0.0292 (15)	0.0012 (11)	0.0093 (11)	0.0024 (11)
C4	0.0246 (15)	0.0439 (17)	0.0376 (17)	0.0060 (12)	0.0046 (11)	0.0013 (14)
C5	0.0207 (14)	0.0578 (19)	0.0425 (18)	−0.0052 (13)	0.0044 (12)	−0.0043 (16)
C6	0.0323 (16)	0.0418 (16)	0.0368 (17)	−0.0130 (13)	0.0126 (12)	−0.0085 (13)
C7	0.0290 (15)	0.0355 (15)	0.0319 (16)	−0.0079 (12)	0.0146 (11)	−0.0076 (13)
C8	0.0224 (13)	0.0252 (14)	0.0273 (14)	0.0002 (11)	0.0084 (10)	−0.0021 (13)
C9	0.0299 (15)	0.0271 (13)	0.0277 (14)	−0.0078 (12)	0.0119 (10)	−0.0036 (13)
C10	0.0274 (16)	0.0276 (14)	0.0301 (16)	−0.0066 (12)	0.0073 (11)	0.0058 (12)
C11	0.0249 (15)	0.0258 (13)	0.0362 (16)	0.0015 (11)	0.0100 (11)	0.0087 (12)
C12	0.0279 (14)	0.0232 (13)	0.0333 (15)	0.0039 (11)	0.0121 (11)	−0.0018 (12)
C13	0.0425 (17)	0.0523 (18)	0.0275 (16)	−0.0021 (14)	0.0105 (13)	−0.0078 (14)
C14	0.0409 (17)	0.0547 (19)	0.061 (2)	−0.0082 (16)	0.0327 (15)	−0.0181 (18)
C15	0.0506 (19)	0.0452 (18)	0.0344 (17)	−0.0140 (15)	0.0189 (13)	0.0004 (15)
C16	0.0299 (16)	0.0473 (19)	0.0361 (17)	−0.0025 (12)	0.0033 (12)	0.0105 (14)

### Geometric parameters (Å, º)

**Table:**

Cl1—C2	1.764 (3)	C8—C9	1.497 (3)
Cl2—C2	1.766 (3)	C8—H8	1.0000
O1—C11	1.218 (3)	C9—C10	1.336 (4)
C1—C2	1.509 (4)	C9—H9	0.9500
C1—C12	1.519 (3)	C10—C11	1.472 (4)
C1—C8	1.523 (4)	C10—C16	1.505 (4)
C1—C3	1.526 (3)	C11—C12	1.516 (4)
C2—C3	1.506 (4)	C12—H12A	0.9900
C3—C13	1.509 (4)	C12—H12B	0.9900
C3—C4	1.529 (4)	C13—H13A	0.9800
C4—C5	1.534 (4)	C13—H13B	0.9800
C4—H4A	0.9900	C13—H13C	0.9800
C4—H4B	0.9900	C14—H14A	0.9800
C5—C6	1.529 (4)	C14—H14B	0.9800
C5—H5A	0.9900	C14—H14C	0.9800
C5—H5B	0.9900	C15—H15A	0.9800
C6—C7	1.543 (4)	C15—H15B	0.9800
C6—H6A	0.9900	C15—H15C	0.9800
C6—H6B	0.9900	C16—H16A	0.9800
C7—C14	1.526 (4)	C16—H16B	0.9800
C7—C15	1.541 (4)	C16—H16C	0.9800
C7—C8	1.579 (4)		
			
C2—C1—C12	117.2 (2)	C1—C8—C7	115.1 (2)
C2—C1—C8	119.0 (2)	C9—C8—H8	106.2
C12—C1—C8	112.4 (2)	C1—C8—H8	106.2
C2—C1—C3	59.49 (16)	C7—C8—H8	106.2
C12—C1—C3	121.2 (2)	C10—C9—C8	125.8 (2)
C8—C1—C3	118.0 (2)	C10—C9—H9	117.1
C3—C2—C1	60.82 (17)	C8—C9—H9	117.1
C3—C2—Cl1	121.74 (18)	C9—C10—C11	119.9 (2)
C1—C2—Cl1	121.15 (19)	C9—C10—C16	122.9 (3)
C3—C2—Cl2	119.2 (2)	C11—C10—C16	117.1 (2)
C1—C2—Cl2	119.59 (18)	O1—C11—C10	121.7 (2)
Cl1—C2—Cl2	108.11 (14)	O1—C11—C12	120.5 (2)
C2—C3—C13	119.9 (2)	C10—C11—C12	117.7 (2)
C2—C3—C1	59.68 (17)	C11—C12—C1	111.6 (2)
C13—C3—C1	120.9 (2)	C11—C12—H12A	109.3
C2—C3—C4	117.6 (2)	C1—C12—H12A	109.3
C13—C3—C4	113.3 (2)	C11—C12—H12B	109.3
C1—C3—C4	115.6 (2)	C1—C12—H12B	109.3
C3—C4—C5	111.3 (2)	H12A—C12—H12B	108.0
C3—C4—H4A	109.4	C3—C13—H13A	109.5
C5—C4—H4A	109.4	C3—C13—H13B	109.5
C3—C4—H4B	109.4	H13A—C13—H13B	109.5
C5—C4—H4B	109.4	C3—C13—H13C	109.5
H4A—C4—H4B	108.0	H13A—C13—H13C	109.5
C6—C5—C4	114.8 (2)	H13B—C13—H13C	109.5
C6—C5—H5A	108.6	C7—C14—H14A	109.5
C4—C5—H5A	108.6	C7—C14—H14B	109.5
C6—C5—H5B	108.6	H14A—C14—H14B	109.5
C4—C5—H5B	108.6	C7—C14—H14C	109.5
H5A—C5—H5B	107.6	H14A—C14—H14C	109.5
C5—C6—C7	118.0 (3)	H14B—C14—H14C	109.5
C5—C6—H6A	107.8	C7—C15—H15A	109.5
C7—C6—H6A	107.8	C7—C15—H15B	109.5
C5—C6—H6B	107.8	H15A—C15—H15B	109.5
C7—C6—H6B	107.8	C7—C15—H15C	109.5
H6A—C6—H6B	107.1	H15A—C15—H15C	109.5
C14—C7—C15	108.0 (2)	H15B—C15—H15C	109.5
C14—C7—C6	109.7 (2)	C10—C16—H16A	109.5
C15—C7—C6	106.7 (2)	C10—C16—H16B	109.5
C14—C7—C8	108.2 (2)	H16A—C16—H16B	109.5
C15—C7—C8	111.9 (2)	C10—C16—H16C	109.5
C6—C7—C8	112.2 (2)	H16A—C16—H16C	109.5
C9—C8—C1	110.0 (2)	H16B—C16—H16C	109.5
C9—C8—C7	112.5 (2)		

## References

[bb1] Agilent (2010). *CrysAlis PRO* . Agilent Technologies Ltd, Yarnton, England.

[bb2] Benharref, A., El Ammari, L., Lassaba, E., Ourhriss, N. & Berraho, M. (2012). *Acta Cryst.* E**68**, o2502. 10.1107/S1600536812032333 PMC3414955 22904942

[bb3] Chekroun, A., Jarid, A., Benharref, A. & Boutalib, A. (2000). *J. Org. Chem.* **65**, 4431–4434. 10.1021/jo991848c 10891148

[bb4] Cremer, D. & Pople, J. A. (1975). *J. Am. Chem. Soc.* **97**, 1354–1358.

[bb5] Dakir, M., Auhmani, A., Ait Itto, M. Y., Mazoir, N., Akssira, M., Pierrot, M. & Benharref, A. (2004). *Synth. Commun.* **34**, 2001–2008.

[bb6] Daoubi, M., Duran -Patron, R., Hmamouchi, M., Hernandez-Galan, R., Benharref, A. & Isidro, G. C. (2004). *Pest Manag. Sci.* **60**, 927–932. 10.1002/ps.891 15382508

[bb7] El Jamili, H., Auhmani, A., Dakir, M., Lassaba, E., Benharref, A., Pierrot, M., Chiaroni, A. & Riche, C. (2002). *Tetrahedron Lett.* **43**, 6645–6648.

[bb8] Farrugia, L. J. (2012). *J. Appl. Cryst.* **45**, 849–854.

[bb9] Flack, H. D. & Bernardinelli, G. (2000). *J. Appl. Cryst.* **33**, 1143–1148.

[bb10] Joseph, T. C. & Dev, S. (1968). *Tetrahedron*, **24**, 3841–3859.

[bb11] Lassaba, E., Eljamili, H., Chekroun, A., Benharref, A., Chiaroni, A., Riche, C. & Lavergne, J.-P. (1998). *Synth. Commun.* **28**, 2641–2651.

[bb12] Plattier, M. & Teiseire, P. (1974). *Recherche*, **19**, 131–144.

[bb13] Sheldrick, G. M. (2008). *Acta Cryst.* A**64**, 112–122. 10.1107/S0108767307043930 18156677

[bb14] Spek, A. L. (2009). *Acta Cryst.* D**65**, 148–155. 10.1107/S090744490804362X PMC2631630 19171970

